# Mapping Social Motivation Networks in Autism Spectrum Disorder: A 3D, Graph-Based Connectomics Analysis

**DOI:** 10.1192/j.eurpsy.2026.10198

**Published:** 2026-07-31

**Authors:** N. Ullman, F. E. Bellomi, F. Petrocchi, C. Caturano, E. Cauzzi, F. Mittoni, L. LaBarbera, G. Loffredo, M. Ribolsi, G. Albergo, P. Soda, M. D’Amelio, G. Vivacqua

**Affiliations:** 1 Microscopic and Ultrastructural Anatomy Laboratory, Campus Bio-medico University of Rome; 2 Molecular Neuroscience Laboratory, Campus Bio-medico University of Rome; 3 Interconsulting services, RINA Services S.p.A; 4 Department of Psychiatry, Policlinico Universitario Campus Bio-medico; 5 Department of Bio-engineering, Campus Bio-medico University of Rome, Rome, Italy

## Abstract

**Introduction:**

Autism spectrum disorder (ASD) spans many symptoms. We focus on those most relevant to a social circuit account: reduced social motivation and initiation, cognitive inflexibility, and heightened sensory reactivity. We examined three interacting systems: a fronto-limbic cortico-striatal–thalamic loop, cerebellar modulation of mesolimbic dopamine, and a rapid subcortical visual route to the amygdala, alongside insular salience. These features are key in functional outcome and caregiver burden, and their improvement is a central target of clinical care. Prior work has connected these systems to motivation, cognitive control, and sensory regulation relevant to these domains. Building on this, we evaluate whether model-specific and convergent alterations at the level of network organization and connectivity strength account for symptom-relevant variance.

**Objectives:**

Build a 3D connectomics dataset of the target areas and test model-specific and convergent circuit alterations relevant to social motivation and salience in ASD.

**Methods:**

We analyzed three mouse models: Fmr1 KO, Shank3b KO, and Tsc1 cKO, with controls. Brains were processed for immunofluorescence with antibody labeling of neuronal classes and substructures, and imaged by confocal microscopy. Tiles were stitched with BigStitcher. Arivis Pro U-Net segmented soma, axon hillock, dendrites, and nuclei to generate voxel masks. Automated 3D reconstruction used Vaa3D APP2 with export as SWC. Traces and volumes were registered to a reference atlas covering the relevant target areas. A NetworkX-based data analysis pipeline converted SWC data to region-level directed graphs and performed group comparisons between each model and matched controls as well as across models. Simulations in MATLAB provided simplified models of axonal excitability, dendritic integration, and synaptic currents to contextualize the network findings.

**Results:**

We assembled a circuit-resolution connectomics dataset across all models. Network analysis showed weaker top-down drive within the fronto-limbic loop with fewer relay connections, reduced cerebellar modulation of mesolimbic control centers (lower hub centrality), and greater fragmentation along a rapid subcortical visual–affective route. The insular network also decoupled from frontal–striatal nodes, yielding a sparser, less integrated social-circuit backbone.

**Conclusions:**

Taken together, the network that supports social valuation, control, and orienting appears less able to drive approach, to adapt when context changes, and to stabilize sensory responses. The transmitter pattern suggests damped catecholaminergic drive and greater inhibitory tone in key targets. This open access 3D, projection-defined map converts those tendencies into quantitative region-to-region measures suited for replication, cross-model comparison, and testing circuit-focused interventions.

**Disclosure of Interest:**

None Declared

